# Supplementary material for: Natural Killer Anti-Tumor Activity Can Be Achieved by In Vitro Incubation With Heat-Killed BCG
Source: Front Immunol. 2021 Feb 23;12:622995. doi: 10.3389/fimmu.2021.622995 (PMC7940681; doi:10.3389/fimmu.2021.622995)
Supplement: Supplementary file 1 [file Presentation_1.pptx]

## Slide 1
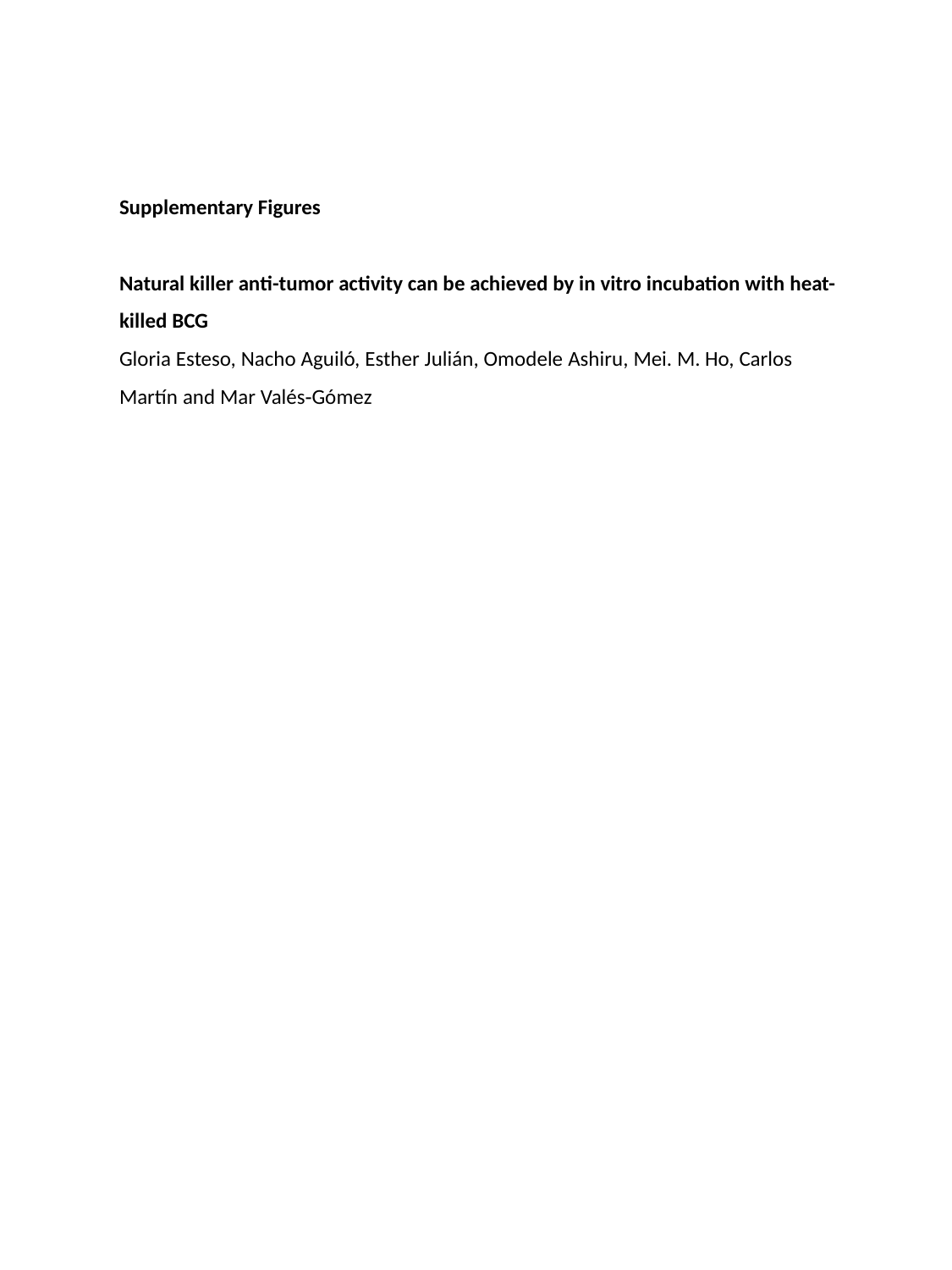

Supplementary Figures
Natural killer anti-tumor activity can be achieved by in vitro incubation with heat-killed BCG
Gloria Esteso, Nacho Aguiló, Esther Julián, Omodele Ashiru, Mei. M. Ho, Carlos Martín and Mar Valés‐Gómez

## Slide 2
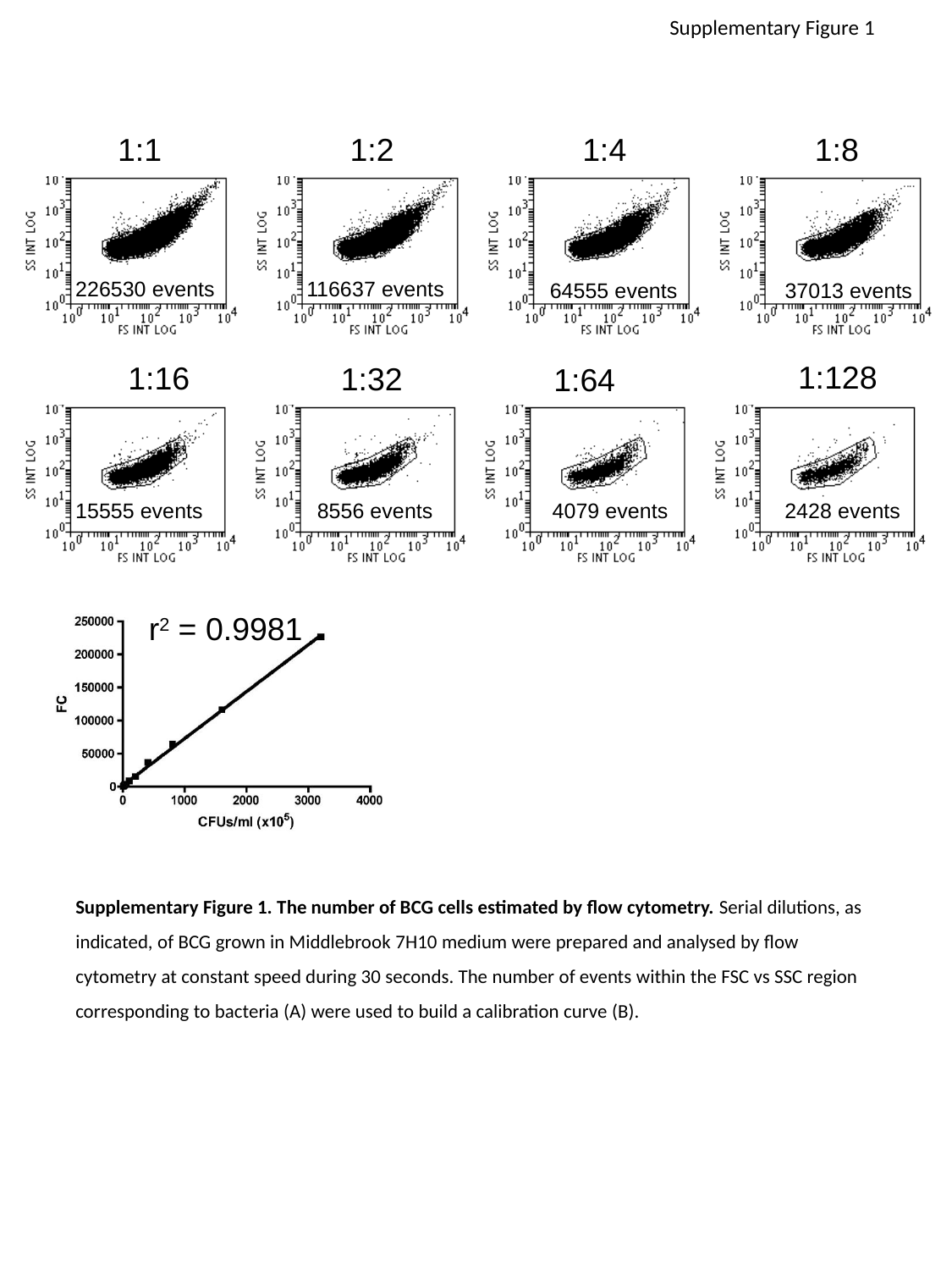

Supplementary Figure 1
1:1
1:2
1:4
1:8
116637 events
226530 events
64555 events
37013 events
1:128
1:16
1:32
1:64
15555 events
8556 events
4079 events
2428 events
r2 = 0.9981
Supplementary Figure 1. The number of BCG cells estimated by flow cytometry. Serial dilutions, as indicated, of BCG grown in Middlebrook 7H10 medium were prepared and analysed by flow cytometry at constant speed during 30 seconds. The number of events within the FSC vs SSC region corresponding to bacteria (A) were used to build a calibration curve (B).

## Slide 3
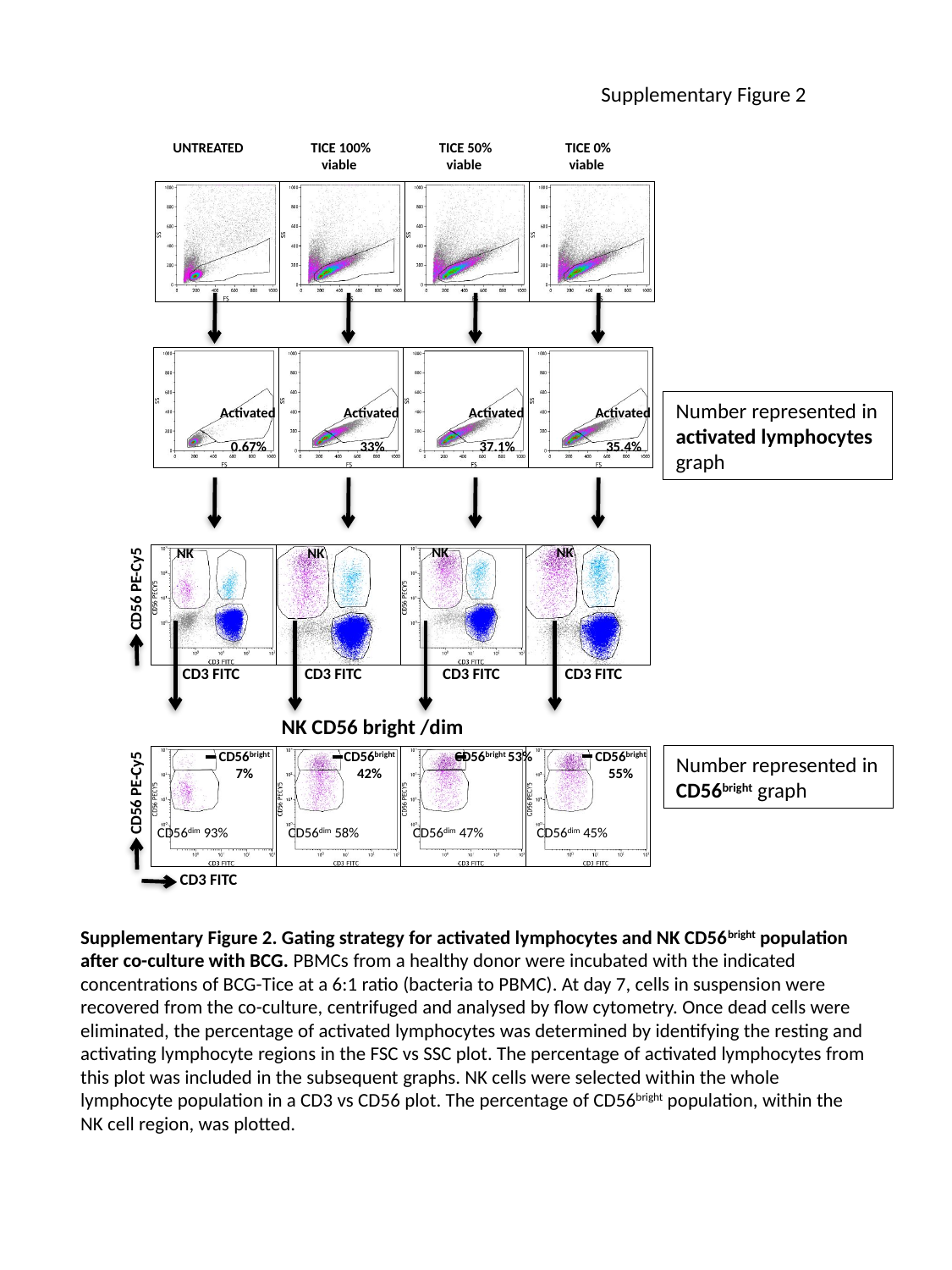

Supplementary Figure 2
UNTREATED
 TICE 100% viable
 TICE 50% viable
 TICE 0%
viable
Activated
0.67%
Activated
33%
Activated
37.1%
Activated
35.4%
Number represented in activated lymphocytes graph
NK
NK
NK
NK
CD56 PE-Cy5
CD3 FITC
CD3 FITC
CD3 FITC
CD3 FITC
NK CD56 bright /dim
CD56bright 7%
CD56bright 42%
CD56bright 53%
CD56bright 55%
CD56 PE-Cy5
CD56dim 93%
CD56dim 58%
CD56dim 47%
CD56dim 45%
CD3 FITC
Number represented in CD56bright graph
Supplementary Figure 2. Gating strategy for activated lymphocytes and NK CD56bright population after co-culture with BCG. PBMCs from a healthy donor were incubated with the indicated concentrations of BCG-Tice at a 6:1 ratio (bacteria to PBMC). At day 7, cells in suspension were recovered from the co-culture, centrifuged and analysed by flow cytometry. Once dead cells were eliminated, the percentage of activated lymphocytes was determined by identifying the resting and activating lymphocyte regions in the FSC vs SSC plot. The percentage of activated lymphocytes from this plot was included in the subsequent graphs. NK cells were selected within the whole lymphocyte population in a CD3 vs CD56 plot. The percentage of CD56bright population, within the NK cell region, was plotted.

## Slide 4
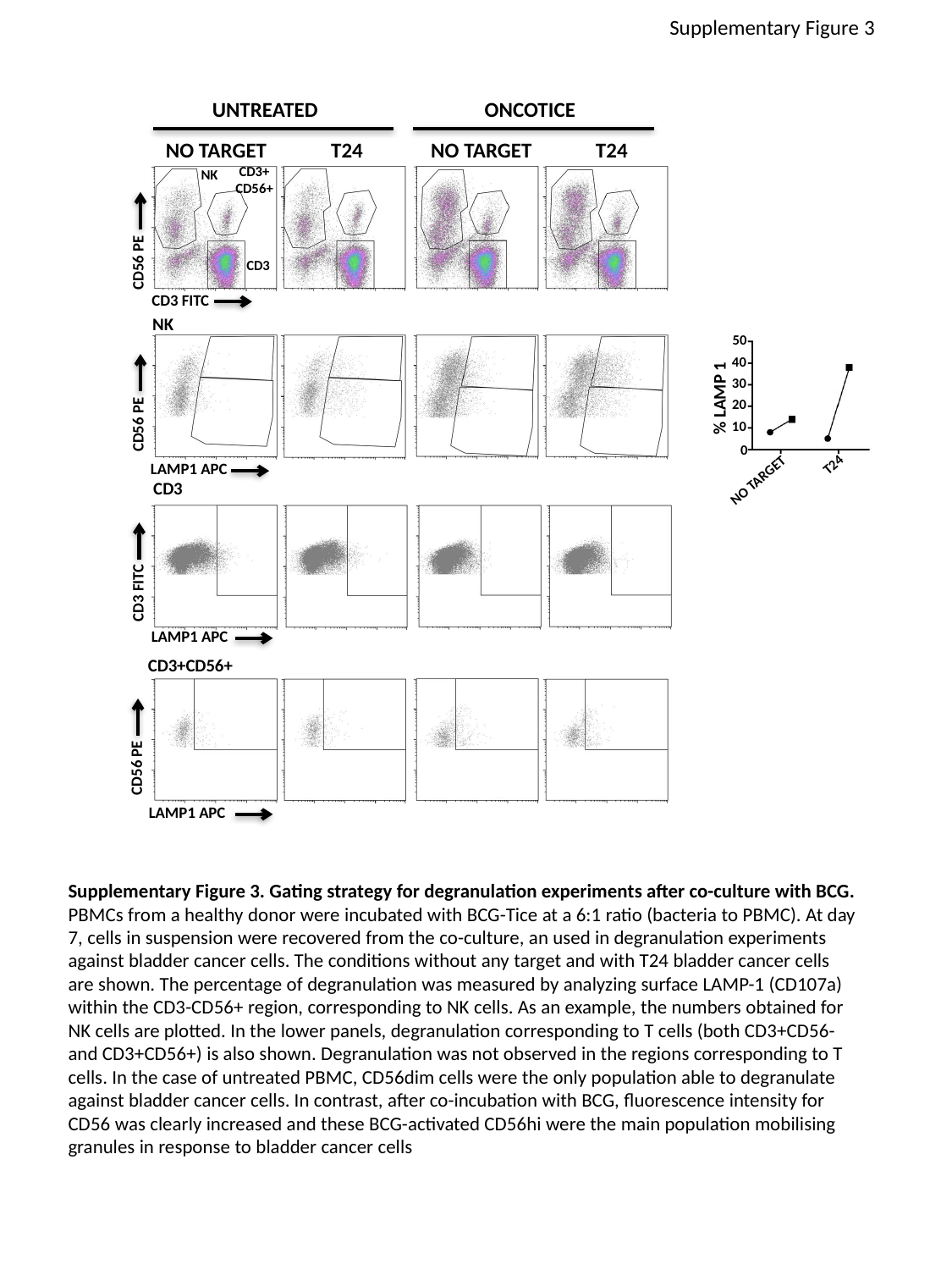

Supplementary Figure 3
UNTREATED
ONCOTICE
NO TARGET
T24
NO TARGET
T24
CD3+
CD56+
NK
CD56 PE
CD3
CD3 FITC
NK
50
40
30
% LAMP 1
20
10
0
T24
NO TARGET
CD56 PE
LAMP1 APC
CD3
CD3 FITC
LAMP1 APC
CD3+CD56+
CD56 PE
LAMP1 APC
Supplementary Figure 3. Gating strategy for degranulation experiments after co-culture with BCG. PBMCs from a healthy donor were incubated with BCG-Tice at a 6:1 ratio (bacteria to PBMC). At day 7, cells in suspension were recovered from the co-culture, an used in degranulation experiments against bladder cancer cells. The conditions without any target and with T24 bladder cancer cells are shown. The percentage of degranulation was measured by analyzing surface LAMP-1 (CD107a) within the CD3-CD56+ region, corresponding to NK cells. As an example, the numbers obtained for NK cells are plotted. In the lower panels, degranulation corresponding to T cells (both CD3+CD56- and CD3+CD56+) is also shown. Degranulation was not observed in the regions corresponding to T cells. In the case of untreated PBMC, CD56dim cells were the only population able to degranulate against bladder cancer cells. In contrast, after co-incubation with BCG, fluorescence intensity for CD56 was clearly increased and these BCG-activated CD56hi were the main population mobilising granules in response to bladder cancer cells

## Slide 5
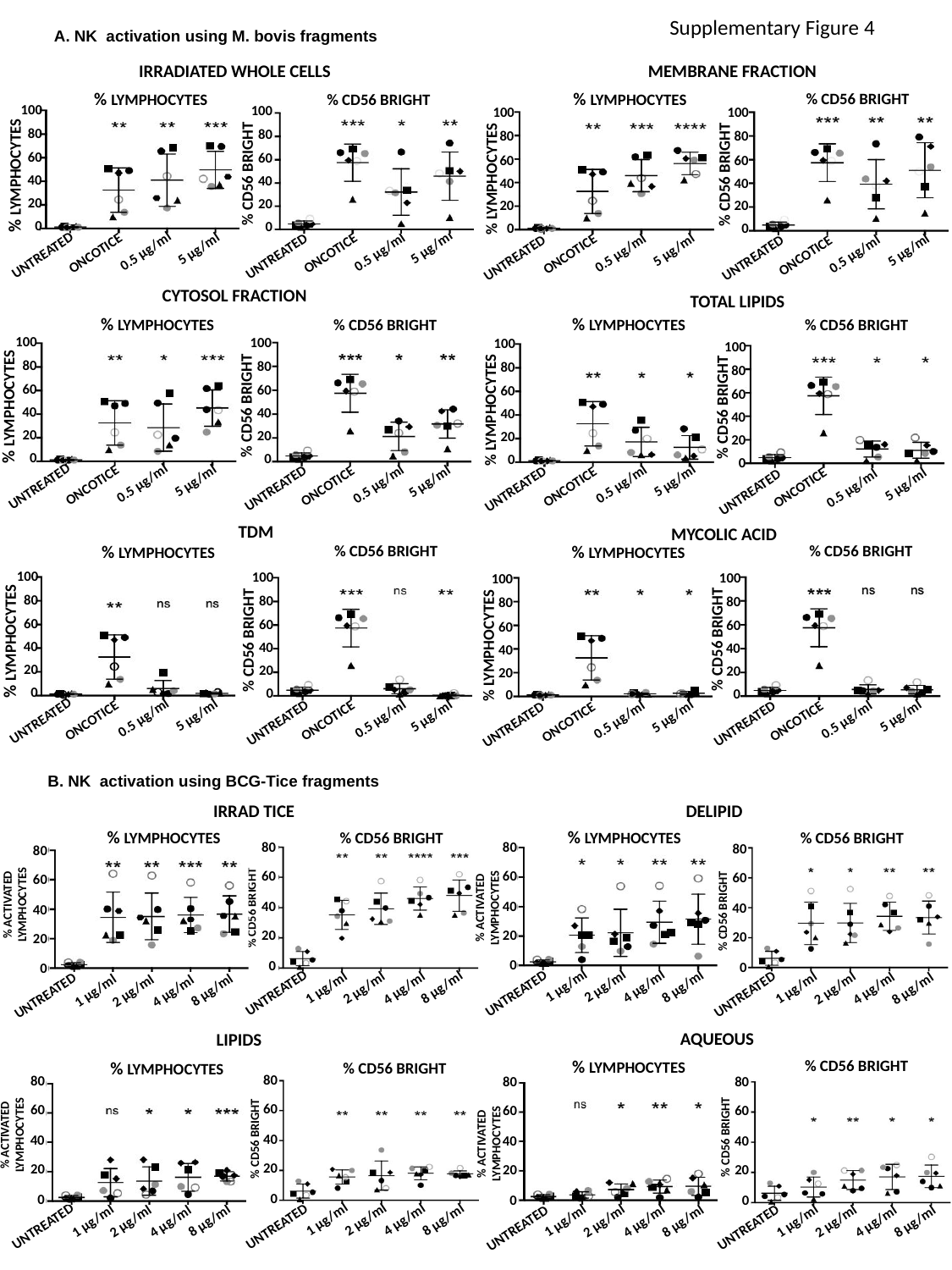

Supplementary Figure 4
A. NK activation using M. bovis fragments
IRRADIATED WHOLE CELLS
100
100
80
80
% CD56 BRIGHT
60
% LYMPHOCYTES
60
40
40
20
20
0
0
0.5 μg/ml
0.5 μg/ml
5 μg/ml
5 μg/ml
UNTREATED
ONCOTICE
UNTREATED
ONCOTICE
% LYMPHOCYTES
% CD56 BRIGHT
MEMBRANE FRACTION
100
100
80
80
% CD56 BRIGHT
60
60
% LYMPHOCYTES
40
40
20
20
0
0
0.5 μg/ml
0.5 μg/ml
5 μg/ml
5 μg/ml
UNTREATED
UNTREATED
ONCOTICE
ONCOTICE
% LYMPHOCYTES
% CD56 BRIGHT
CYTOSOL FRACTION
100
100
80
80
% CD56 BRIGHT
% LYMPHOCYTES
60
60
40
40
20
20
0
0
0.5 μg/ml
0.5 μg/ml
UNTREATED
5 μg/ml
5 μg/ml
ONCOTICE
UNTREATED
ONCOTICE
% LYMPHOCYTES
% CD56 BRIGHT
TOTAL LIPIDS
100
100
80
80
% CD56 BRIGHT
60
% LYMPHOCYTES
60
40
40
20
20
0
0
0.5 μg/ml
5 μg/ml
ONCOTICE
0.5 μg/ml
UNTREATED
5 μg/ml
ONCOTICE
UNTREATED
% LYMPHOCYTES
% CD56 BRIGHT
TDM
100
100
80
80
% CD56 BRIGHT
% LYMPHOCYTES
60
60
40
40
20
20
0
0
0.5 μg/ml
0.5 μg/ml
UNTREATED
5 μg/ml
5 μg/ml
ONCOTICE
UNTREATED
ONCOTICE
MYCOLIC ACID
100
100
80
80
% CD56 BRIGHT
60
60
% LYMPHOCYTES
40
40
20
20
0
0
0.5 μg/ml
0.5 μg/ml
5 μg/ml
5 μg/ml
UNTREATED
UNTREATED
ONCOTICE
ONCOTICE
% LYMPHOCYTES
% CD56 BRIGHT
% LYMPHOCYTES
% CD56 BRIGHT
B. NK activation using BCG-Tice fragments
IRRAD TICE
% LYMPHOCYTES
% CD56 BRIGHT
80
80
60
% CD56 BRIGHT
60
% ACTIVATED
LYMPHOCYTES
40
40
20
20
0
0
8 μg/ml
1 μg/ml
2 μg/ml
4 μg/ml
8 μg/ml
1 μg/ml
2 μg/ml
4 μg/ml
UNTREATED
UNTREATED
DELIPID
% LYMPHOCYTES
% CD56 BRIGHT
80
60
% ACTIVATED
LYMPHOCYTES
40
20
0
1 μg/ml
2 μg/ml
4 μg/ml
8 μg/ml
UNTREATED
80
60
% CD56 BRIGHT
40
20
0
1 μg/ml
2 μg/ml
4 μg/ml
8 μg/ml
UNTREATED
 AQUEOUS
% LYMPHOCYTES
% CD56 BRIGHT
80
60
% ACTIVATED
LYMPHOCYTES
40
20
0
1 μg/ml
2 μg/ml
4 μg/ml
8 μg/ml
UNTREATED
80
% CD56 BRIGHT
60
40
20
0
1 μg/ml
2 μg/ml
4 μg/ml
8 μg/ml
UNTREATED
LIPIDS
% LYMPHOCYTES
80
80
% CD56 BRIGHT
60
60
% ACTIVATED
LYMPHOCYTES
40
40
20
20
0
0
1 μg/ml
2 μg/ml
4 μg/ml
8 μg/ml
8 μg/ml
1 μg/ml
2 μg/ml
4 μg/ml
UNTREATED
UNTREATED
% CD56 BRIGHT

## Slide 6
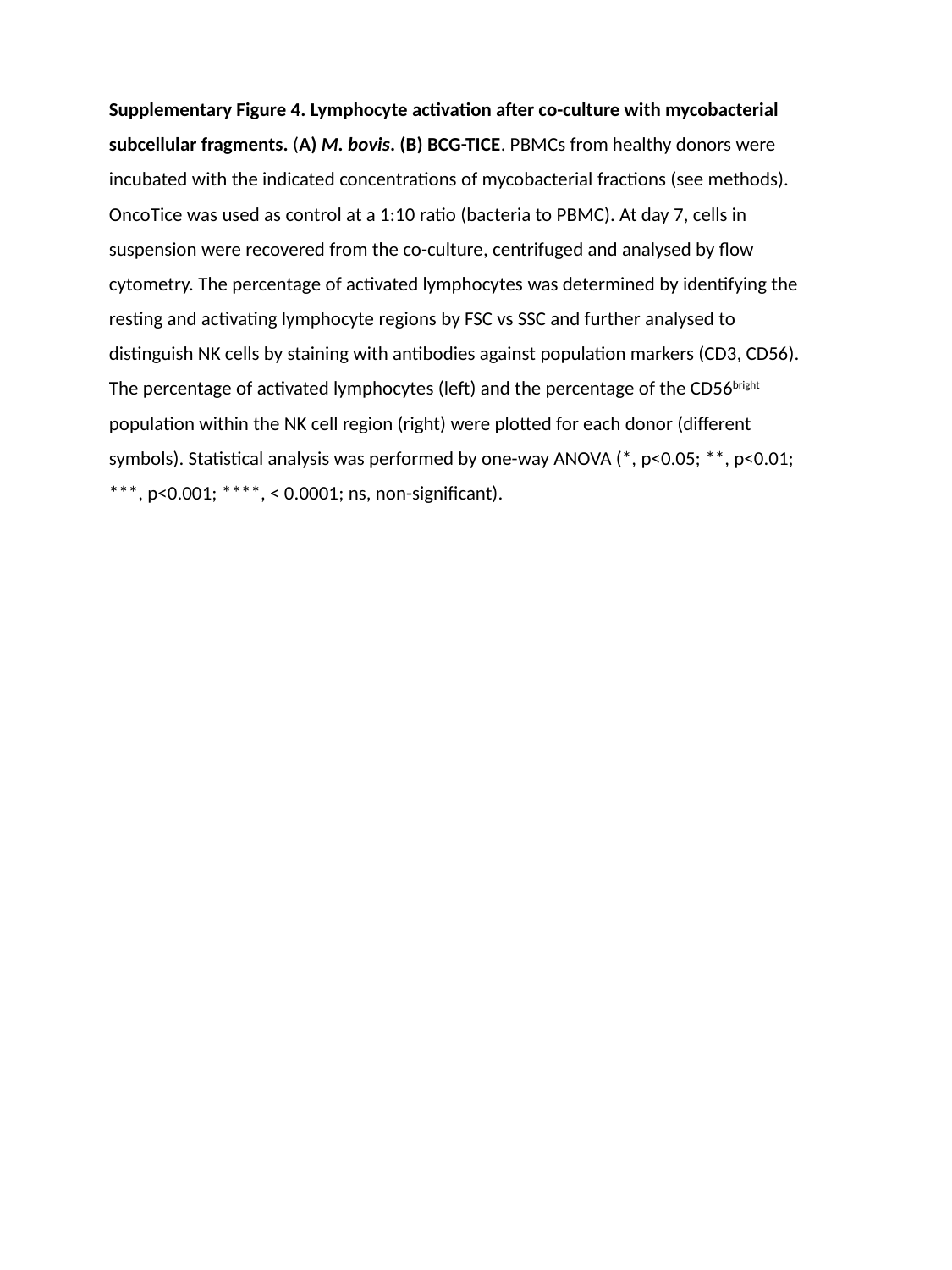

Supplementary Figure 4. Lymphocyte activation after co-culture with mycobacterial subcellular fragments. (A) M. bovis. (B) BCG-TICE. PBMCs from healthy donors were incubated with the indicated concentrations of mycobacterial fractions (see methods). OncoTice was used as control at a 1:10 ratio (bacteria to PBMC). At day 7, cells in suspension were recovered from the co-culture, centrifuged and analysed by flow cytometry. The percentage of activated lymphocytes was determined by identifying the resting and activating lymphocyte regions by FSC vs SSC and further analysed to distinguish NK cells by staining with antibodies against population markers (CD3, CD56). The percentage of activated lymphocytes (left) and the percentage of the CD56bright population within the NK cell region (right) were plotted for each donor (different symbols). Statistical analysis was performed by one-way ANOVA (*, p<0.05; **, p<0.01; ***, p<0.001; ****, < 0.0001; ns, non-significant).
